# The effects of screen light filtering software on cognitive performance and sleep among night workers

**DOI:** 10.15171/hpp.2019.32

**Published:** 2019-08-06

**Authors:** Reza Kazemi, Negar Alighanbari, Zahra Zamanian

**Affiliations:** ^1^Ergonomics Department, School of Health, Shiraz University of Medical Sciences, Shiraz, Iran; ^2^School of Health, Shiraz University of Medical Sciences, Shiraz, Iran

**Keywords:** Cognitive function, Night shift work, Sleepiness, Sleep

## Abstract

**Background:** Previous studies have reported impaired performance, sleepiness and sleep deprivation among night workers. The purpose of this study was to investigate the effect of color screen Light Filtering software on cognitive performance, alertness and sleep quality among night shift operators of a medical emergency operations center.

**Methods:** This field trial interventional study was carried out among 30 nightshift operators of shiraz emergency control center. The baseline assessments were carried out under the existing computer screen light conditions in the week preceding the installation of f.lux software. The same measurements were repeated again 4 weeks after installing the software. The cognitive performance of the participants was measured using continuous performance test (CPT) and n-back, while their sleep quality was assessed through Pietersburg Sleep Quality Index (PSQI). Further, to assess their subjective and objective alertness, Stanford sleepiness index and go/nogo test were used, respectively.

**Results:** The results of this study showed that Screen Light Filtering software significantly increased subjective (P<0.001) and objective alertness (P<0.05). Additionally, the performance of the working memory (P=0.008) and sleep quality (P=0.008) improved significantly after the intervention.

** Conclusion:** The results revealed that using Screen Light Filtering software is an effective and low-cost method to improve sleep quality and cognitive performance since it filters the short wavelength part of the spectrum and helps body adaptation.

## Introduction

Numerous studies have reported impaired performance, sleepiness and sleep deprivation among night workers.^1^ The results of field and experimental studies show that night work interferes with the circadian and homeostatic regulation of sleep.^2,3^ Lack of sufficient sleep results in sleepiness and low alertness. It can also impair cognitive performance, such as impaired attention, working memory which affects other functions, such as long-term memory and decision-making.^3,4^ Such a decline in neuro-cognitive performances may in turn cause damages arising from fatigue and human error.^5^

Given the nature of some jobs and societal needs in today’s world, a considerable number of workers are engaged in shift works. Operators employed in medical emergency operation centers are no exception in this regard. They have to be present at workplace day and night to offer necessary health services to patients prior to transferring them to hospitals. Indeed, they are the first group of health service providers who are in direct contact with patients’ families, providing initial information about patients’ status. Thus, people working in medical emergency services should conduct a speedy initial diagnosis of the patient’s disease and make instant decisions on which hospital they should transfer the patient to. Under such circumstances, nurses in medical emergency centers require accuracy, speed, proper mental performance and high alertness for doing the tasks properly.^6^

Numerous solutions have been recommended for improving the performance and increasing the alertness of night shift employees. Increasing the illumination and color temperature of light, using melatonin incentives such as coffee drinks and proper plan of night shifts are some of these solutions.^2,7^ Spectral variations of work environment lighting have been recently noticed as a workable alternative.^8^ Research findings have shown that spectral specifications of lighting such as the color temperature and wavelength of light can create non-visual effects, hence influencing the quality of sleep and circadian rhythm.^2,5,8^

Scientific evidence has indicated that due to the high sensitivity of intrinsically photosensitive retinal ganglion cells (ipRGCs) to short wavelength light, the light with short wavelength has a more profound impact on melatonin suppression and change of circadian phase than the one with longer wavelength.^9-11^ Moreover, it has been stated in previous studies that although the light of short wavelength strengthens alertness and cognitive performance,^9,12^ through activating light receivers, known as ipRGC, these wavelengths can stop the secretion of melatonin hormone and disturb the 24-hour cycle of body day and night rhythm as well as sleep process in the resting time.^13^ Study findings have also corroborated that suppressed secretion of melatonin hormone may cause strong negative effects on body health such as breast cancer, colon cancer and prostate cancer.^14-16^

Studies have shown that unlike lights with short wavelength, long-wavelength lights can also maintain the natural rhythm of body during night, maintain alertness and cognitive performance without suppressing melatonin hormone^13,17,18^ and contribute to the improvement of sleep quality.^19,20^ As such, some researchers have argued in favor of filtering and/or reducing the amount of short-wavelength light emitted by computer screens to improve alertness and quality of sleep. There are various software and hardware solutions to filter and reduce the light waves emitted from the computer screen including using glasses which filter the light and prevent the light of short-wavelength from reaching the eye, using installable physical filters on computer monitor^17,20,21^ and installing filtering software programs such as f.lux software. f.lux has been used in different studies to filter the light of short wavelength especially the blue one,^17,21^ the results of which showed the positive impact of this software on cognitive performance and alertness. These studies, however, have a number of limitations; firstly, they have been conducted in experimental, rather than real settings. Secondly, the effect of this software on the performance and alertness of various groups of people and night shift workers has not been investigated. Moreover, as mentioned before, there are contradictory results regarding the impact of filtering or strengthening the lights with short waves on performance and alertness. Addressing the existing doubts, the aim of this study was to investigate the effect of using filtering software with short wave light on the performance and alertness of night shift workers in a real environment. Given that a large bulk of the duties of emergency service operators is performed while they face computer screens, intervention on these devices may have immediate and cheaper effects.

## Materials and Methods

### Participants

The participants included 30 night shift operators of Shiraz Medical Emergency Operation center. They were all females, with a mean (SD) age of 29.97 (5.47) years and a mean night-shift work experience of 5.7 (4.24) years. All participants were non-smokers and were instructed to refrain from consuming caffeine or alcohol during the 12-hour period preceding the experiments. All the participants met the following criteria: (1) none of them used hypnotic drugs; (2) no one suffered from any psychological/main systematic illness or sleep disorder; and (3) none of the participants was “extreme late” (MEQ score > 70 ) or “extreme early” (MEQ score <30) type, according to their responses to the Munich Chronotype Questionnaire.^22^ In order to observe ethical issues, all participants provided informed consent prior to the experiment.

### Study design and procedure

**Figure 1:**
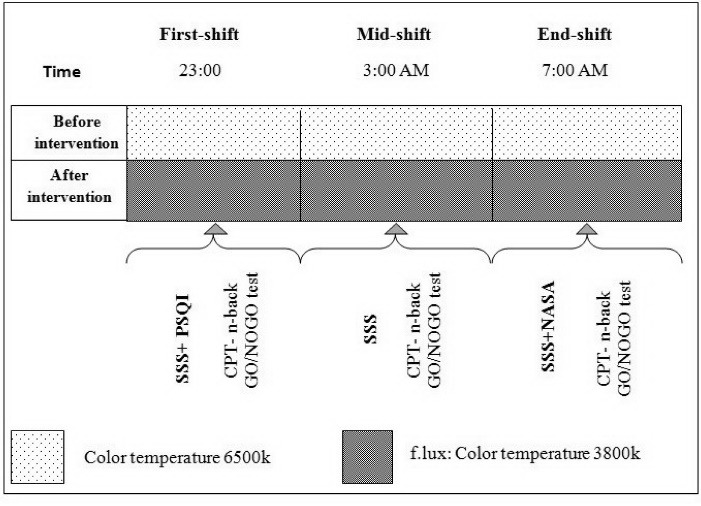
Schematic representation of the study protocol.

The study was a field trial intervention in design, with duration of 4 weeks, and was conducted in two stages from September 23, 2017 through November 21, 2017. All the participants were almost similar in terms of the intensity of environment light exposure and staff responsibilities. Furthermore, their shift-work schedule consisted of 2 night shifts, 3 day shifts, and 2 days off, with each shift lasting for 8 hours. Participants were excluded from the study if their work schedule was not mentioned above. Since the study aimed at investigating the influence of f.lux software during night shifts, all the assessments were performed only during these shifts. In addition, in order to eliminate the adaptation effect to doing several night shifts in a row, both assessments were carried out during the second consecutive night shift. The baseline assessments were carried out under the existing computer screen light conditions in the week preceding the installation of f.lux software. The same measurements were repeated again 4 weeks after installing the software. The software installation took place over the weekend, before night shifts; assessments were conducted three times with 4-hour intervals (at the beginning, in the middle, at the end) during the shift (Figure 1). At each stage, the working memory, alertness, vigilance and attention was evaluated. And at the end, shift NASA-TLX was evaluated. The day before the study, the participant’s became familiar with the assessment tools.

### f.lux software

f.lux (v4.104) is a free software program (https://justgetflux.com/), developed for computers and other iDevices, unobtrusively alters light spectrum emitted from screens according to clock time. The screen remains unchanged throughout the day (6500 K, peak ɻ= 453 nm); however, from late evening until early morning, f.lux adjusts the screen light to that of natural light, using warmer colors, such as red and orange (3800 K, peak ɻ= 598 nm; used in the present study), instead of blue/green light used throughout the day.^17^

### Mental workload 

The NASA-TLX, one of the most widely used instruments to assess overall subjective workload, is a multidimensional instrument consisting of 6 subscales: Mental Demand (MD), Physical Demand (PD) and Temporal Demand (TD), Frustration (FR), Effort (EF), and Performance (PE) (see Supplementary file 1 for the NASA TLX). The six scales are combined to create an overall workload scale (0–100). The psychometric properties of Persian version of NASA-TLX were reported by Mohammadi et al.^23^

### Working memory

N-back test is often used to measure working memory capacity and to assess the effect of sleep deprivation on working memory.^24,25^ In the present study, the computer type and n = 1 was used since it has been demonstrated that 1-back test is more sensitive for individuals who have to deal with sleep deprivation. The test includes playing 120 numbers (one at a time) in the center of the computer screen for 5 minutes, with 1500 millisecond intervals. Participants are invited to make a comparison between the last number which appears on the screen and the one displayed before that. If the two consecutive numbers are the same, participants should immediately press the answer button on the keyboard. In this test, the dependent variables involve the number of correct answers and reaction time.^24^

### Continuous performance test 

Continuous performance test (CPT) was used as a measure of sustained attention.^26^ There were 150 Persian numbers as stimuli. Out of this, 30 (20%) were designated as target stimuli and 120 (80%) as non-target stimuli. The attention of participants was determined by measuring their reaction time and calculating the percent of errors during the test.^27,28^

### Go/no go task

The go/no go task is an objective measure of cognitive alertness.^17^ The 4-min computerized task displayed a series of single black letters (either ‘‘B’’ or ‘‘E’’) on a white background using an Acer laptop.

Each letter was displayed for 0.216 seconds and the blank inter-trial interval time varied randomly between 1300 and 1700 ms. The participants responded only to the letter ‘‘B’’ by pressing the spacebar. The response time window was between 150 ms (to stop anticipated responses) and 1500 ms. After 500 ms, a 440-Hz tone sounded for 475 ms to encourage participants to respond. The letter ‘‘E’’ appeared randomly at a frequency of 4 in every 20 letters displayed. The average reaction time (ms), and correct ‘‘no go’’ responses (%) were recorded.

### Stanford Sleepiness Scale

Stanford Sleepiness Scale was used as a subjective measure of alertness and sleepiness.^29^ It is a scale assessing sleepiness on a 1*–*7 scale (1 being the most alert and 7 lost struggle to remain awake.^30^ The internal consistency of the Stanford Sleepiness Scale in this current study was 0.82 (See Supplementary file 1).

### Pittsburgh Sleep Quality Index

Pittsburgh Sleep Quality Index (PSQI) was used to measure sleep quality along 7 subscales including subjective sleep quality, sleep-onset latency, sleep duration, habitual sleep efficiency, sleep disturbances, use of sleeping medication sleep deprivation and daytime dysfunction.^31^ Component scores range from 0 to 3 and are summed to obtain a global score, which ranges from 0 to 21. Higher scores suggest greater sleep disturbance; a global score more than 5 suggests a significant disturbance. As indicated in previous research, the validity and reliability of the Iranian version are 0.86 and 0.89, respectively.^32^ The sleep quality of the participants was assessed before and after the intervention (installingf.lux).

### Statistical analysis

IBM SPSS Statistics software version 20 (IBM Corp., Armonk, NY) was used for data analysis. The Kolmogorov-Smirnov test was conducted to evaluate the normality of the data distribution. The effects of the intervention on cognitive performance and alertness during the work shift were investigated using mixed-model ANOVAs. For further analysis, paired *t* test was carried out for pairwise comparisons of the parameters before and after the intervention and Wilcoxon test was used (instead of paired *t* test) in the case of data that were not normally distributed. Also, the effect of intervention on mental work load and sleep quality were investigated using *t* test. The significance level was considered to be at 0.05.

## Results

The participants included 30 night shift operators of Shiraz Medical Emergency Operation center. They were all females, with a mean (SD) age of 29.97 (5.47) years and a mean night-shift work experience of 5.7 (4.24) years.

### Mental workload

**Figure 2:**
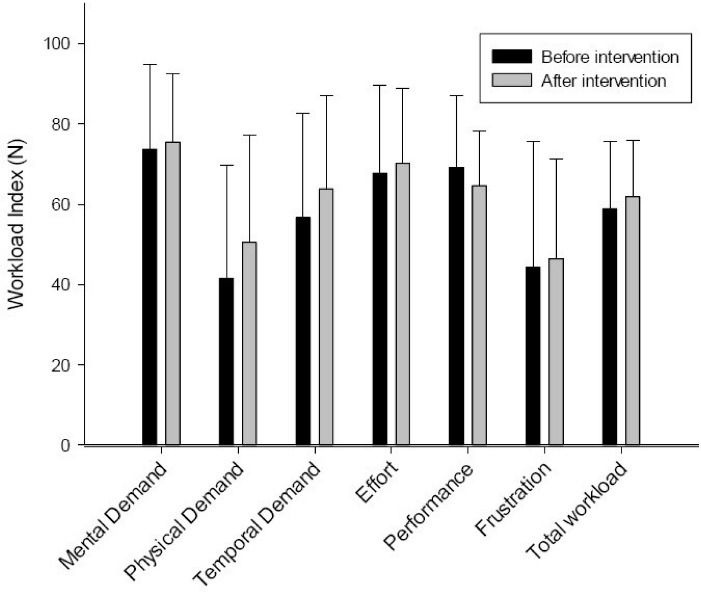
Workload comparison between pre-intervention and post-intervention stages.

As shown in Figure 2, the results of paired samples t-test showed that there was no significant difference in the overall workload between pre-intervention and post-intervention stages, so the participants were relatively the same in their workload before and after the intervention (*P* > 0.05).

### Working memory test (n-back task)

**Figure 3:**
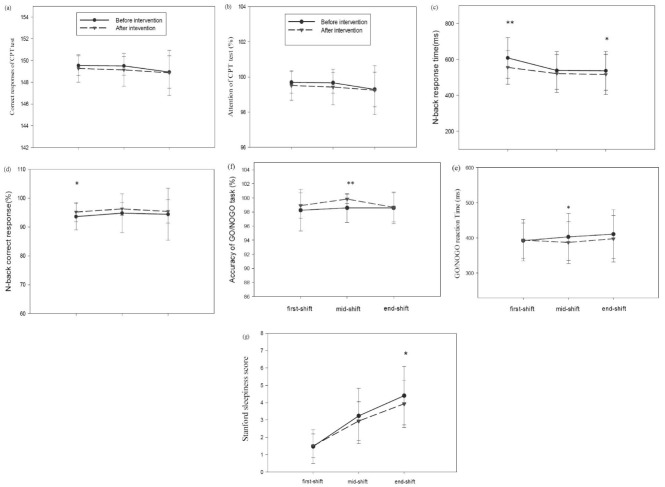
Effects of filtering short wavelengths from computer screen light during night shifts on cognitive performance, subjective and objective measures of alertness. The data points are the means and standard errors for : (a)response time in CPT(ms); (b)percentage of attention in CPT test; (c) n-back response time; (d) correct response percentage of the n-back test; (e) GO/NOGO reaction time; (f)percentage of response accuracy in GO/NOGO task and (g) Stanford sleepiness score, under baseline (black filled circles) and intervention (black filled triangles) conditions over three time points of Computer use at night shift. The aster (* *P* < 0.01 and **P < 0.001) denotes a significant difference between the baseline and intervention condition (f.lux installation).

The effect of intervention on the reaction time of the n-back test (F (1, 29) = 8.59, *P* = 0.007) was significant (Figure 3c). In particular, the results of paired samples *t* test showed that, at the beginning of the work shift, the reaction time (*P* = 0.008) reduced considerably after the intervention. The effects of time (F (1.23, 35.59) = 11.15, *P* = 0.001) and intervention– time interaction (F (2, 58) = 3.75, *P* = 0.03) were also significant.

The effect of intervention on the percentage of correct response to n-back test was significant (F (1, 29) = 4.45, *P* =0.04) (Figure 3d). The results of paired samples *t* test indicated that, at the beginning of the work shift, the percentage of correct response increased significantly (*P* = 0.002) after the intervention. On the contrary, the effects of time (F (1.34, 38.75) = 0.31, *P* = 0.65) and intervention – time interaction (F (1.12, 32.44) = 0.70, *P* = 0.42) were not significant.

### Continuous Performance Test

The effect of intervention on the reaction time of CPT test (F (1.29) = 0.22, *P* = 0.65) was not significant, but the effect of time (F (2, 58) = 8.50, *P* = 0.001) was statistically measurable (Figure 3a). Thus, the percentage of sustained attention decreased significantly towards the end of the work shift. On the other hand, the effect of intervention-time interaction (F (2, 58) = 1.27, *P* = 0.29) was not significant.

On the other hand, the effect of intervention on sustained attention of CPT test (F (1, 29) = 1.56, *P* = 0.22) was not significant. Nonetheless, the effect of time (F (1.65, 47.88) = 4.86, *P* = 0.02) was found to be significant (Figure 3b), meaning that the percentage of sustained attention decreased significantly towards the end of the work shift. At the same time, the intervention-time interaction effect (F (2, 58) = 0.09, *P* = 0.91) was not significant.

### Objective alertness

The effect of intervention on the reaction time of Go / no go test (F = 1.29) = 3.44, *P* = 0.07) and the effect of time F (1.49, 43.27) = 1.44, *P* = 0.25) were not significant (Figure 3e). However, the effect of intervention–time interaction was found to be statistically measurable (F (1.62.46.98) = 3.84, *P* = 0.04).

The effect of intervention on the response accuracy of Go/no go test (F (1.29) = 9.47, *P* = 0.005) was significant (Figure 3f). As the results of paired samples t-test show, this difference was significant in the middle of the work shift. After the intervention, the percentage of response accuracy increased significantly in the middle of the work shift. In contrast, the effects of time (F (1.64.47.60) = 1.42, *P* = 0.25), and intervention-time interaction (F (2.58) = 2.73, *P* = 0.07) did not reach statistical clout.

### Subjective alertness

Although the effect of intervention (F (1.29) = 3.78, P = 0.062) on the degree of subjective alertness and sleepiness (Stanford test) was not significant, the effect of time (F (1.31, 38.01) = 94.67, *P* < 0.001) was significant (Figure 3g). Hence, the degree of sleepiness increased significantly towards the end of the work shift. Also, the effect of intervention-time interaction was significant (F (2, 58) = 4.11, *P* = 0.02) so that the effect of the intervention was different at the end of the work shift in comparison with other times. Accordingly, a separate paired sample *t* test was used at the end of the work shift, indicating a significant difference. After the intervention, the degree of sleepiness declined significantly at the end of the work shift.

### Sleep quality

The final score of sleep quality and subscales of Pittsburgh Sleep Quality Index improved significantly after the intervention (Table 1).

**Table 1:** Comparing of mean and standard deviation of Sleep Quality Index before and after intervention

**Variable**	**Pre-intervention** **Mean (SD)**	**Post-intervention** **Mean (SD)**	**Range of score**	***P*** ** value**
Subjective sleep quality	1.17 (0.87)	0.97 (0.56)	0-3	0.034^a^
Sleep latency	1.73 (0.88)	1.13 (0.82)	0-3	0.001^a^
Sleep duration	0.90 (1.12)	0.6 (0.86)	0-3	0.013^a^
Sleep efficiency	1.00 (1.02)	0.83 (1.08)	0-3	0.450^a^
Sleep disturbances	1.00 (0.59)	0.73 (0.45)	0-3	0.005^a^
Daytime dysfunction	1.03 (1.16)	0.67 (0.76)	0-3	0.010^a^
Total sleep quality score	7.57 (3.90)	5.03 (2.98)	0-21	0.001^b^

^a^ Wilcoxon test; ^b^ Paired *t* test.

## Discussion

Shift workers, especially night shift workers, are less alert and efficient due to irregularity of their sleep and awakening cycle and circadian rhythm. This may cause irreparable human errors in sensitive situations such as the operators of emergency command room, which is of a vital role in patient safety. In this connection, color temperature and light wavelength are considered as the proper and ergonomic action because of their role in empowering visual power and non-visual effects such as tuning the circadian rhythm as well as improving cognitive performance, alertness and the quality of sleep.

The results of this study showed that f.lux software improves the quality of sleep, reducing drowsiness in night shift and increasing cognitive performances with dynamic change of the temperature of computer screen color from 6500 K in base mode to the color temperature of 3800 K through filtering light short wavelength.

### Cognitive performance and alertness 

In this study, f.lux software improved the cognitive performance, including sustainable attention and working memory, of night shift participants. These results are in line with those obtained in other studies. Hoffmann et al, for example, showed that the light with low color temperature (long light wavelength) does not suppress melatonin, leading to maintenance of performance and alertness.^18^ The results of the current study are also in line with the study of Canazei et al, which found no significant difference between cognitive performance (sustainable attention and working memory) in various light conditions with different color temperature of 4667, 3366 and 2116 K.^8^ Likewise, the results of this study showed that the light with reduced wavelength will not affect cognitive performance destructively.^33^ This may be attributed to the stimulation of a part of the limbic system, known as Amygdala, which is of an important role in learning and memory and, apart from suppressing melatonin secretion, it increases alertness and performance.^34^ Yet, the results of this study are in stark contrast with the findings of Motamedzadeh et al, who showed that increasing the temperature of environment lighting from 2000 K to 17 000 K improves cognitive performance and alertness in night shift workers.^35^

To justify the consistency of the results of this study with some of the studies as well as their contradiction with others, alertness and cognitive performance are improved through two different paths: firstly, stimulating a part of the limbic system, known as Amygdala, which is of an important role in learning and memory and, apart from suppressing melatonin secretion, it increases alertness and performance. Secondly, suppressing melatonin as sleeping hormone that short light wavelength improves alertness and performance. Given that suppressing melatonin negatively affects the health, the first path becomes safer.

### Sleep quality 

In the current study, the quality of sleeping was influenced by f.lux software. More specifically, a significant rise in sleep quality was observed as a result of changing the color temperature of screen from color temperature of 6500 kelvins (before the intervention) to 3800 K. Many studies have recently shown that light emitted at night from electronic instruments, especially computer LED monitor, reduces the sleeping period and delays the onset of sleeping and awakening because such a light has short wavelengths.^36-38^ Moreover, many studies have shown that filtering the short wavelength light of the computer monitor at night improves sleeping quality and tunes circadian rhythm.^19,20,39^ A new study by Ostrin et al showed that the emitted short wavelength light from monitors can play a role in the high prevalence of sleeping disorders. They demonstrated that, through filtering short waves by special glasses three hours before sleeping, 58% of blood melatonin increased and their quality of light significantly improved compared with the past,^39^ a finding which is in agreement with our results. The changes in melatonin level were not evaluated in this study. Nonetheless, this result can be interpreted in the light of previous studies. Specifically, given the high sensitivity of light receivers, known as ipRGCs, to light short wavelength in the range of (460-480 nanometer), it is activated through encounter with short wavelength light, leading to suppressed secretion of melatonin hormone, sleep disorder and reduced sleep quality.^4,38,40^ On the contrary, given the lower sensitivity of light receivers to low color temperature and long wavelength of light, melatonin hormone secretion is not suppressed, which contributes to sleep quality.^39^

### Limitations 

In this study, all participants were female in the same age range. Therefore, gender and age differences were ignored in examining the effect of screen light filtering software. Cognitive performance appears to be particularly vulnerable to the effects of advancing age and gender, producing various effects on alertness and sleep quality. Therefore, the influence of age and gender on the function of screen light filtering software should be evaluated in future studies.

Also, a 24-hour assessment of circadian rhythm marker was not conducted. Hence, future studies may investigate circadian profile of melatonin rhythm after installing the screen light filtering software. The measures in the present study were carried out only among night workers engaged in12-hour work shift patterns with fast rotation. It is suggested that future studies examine visual and non-visual effects of screen light filtering software on work shift patterns with different rotations and working hours.

## Conclusion

The results of this study generally showed that f.lux software, improves cognitive performance, alertness and the quality of sleeping in night shift workers. Given that the software filters light waves of short wavelength, with more suppression on the secretion of melatonin as a required strong antioxidant of body, it is a safer and more affordable solution for improving the performance and alertness in night shift workers. It can also be used as an ergonomic solution recommended for improving the performance and reducing human error in nigh shift workers of sensitive environments.

## Ethical approval

This study was approved by the Shiraz University of medical sciences ethics committee (IR.SUMS.REC.1396.38).

## Competing interests

The authors declare that they have no competing interests.

## Funding

Shiraz University of Medical Sciences supported the stady (grant No. 13988).

## Authors’ contributions

RK and NA were involved in study conceptualization, data collection and manuscript revising; ZZ was involved in study conceptualization, statistical analyses, and manuscript writing.

## Acknowledgments

The authors of this article would like to appreciate the financial support provided by Shiraz University of Medical Sciences.

## Supplementary Materials


Supplementary file 1 contains Stanford Sleepiness and NASA-TLX Mental Workload Rating Scales.
