# Supplementary material for: The effects of screen light filtering software on cognitive performance and sleep among night workers
Source: Health Promot Perspect. 2019 Aug 6;9(3):233–40. doi: 10.15171/hpp.2019.32 (PMC6717920; doi:10.15171/hpp.2019.32)
Supplement: Supplementary file 1 — contains Stanford Sleepiness and NASA-TLX Mental Workload Rating Scales. [file hpp-9-233-s001.pdf]

Supplementary file 1

Stanford Sleepiness Scale

Rate your alertness at different times during the day.

**Degree of Sleepiness**	**Scale Rating**
Feeling active, vital, alert, or wide awake	1
Functioning at high levels, but not at peak; able to concentrate	2
Awake, but relaxed; responsive but not fully alert	3
Somewhat foggy, let down	4
Foggy; losing interest in remaining awake; slowed down	5
Sleepy, woozy, fighting sleep; prefer to lie down	6
No longer fighting sleep, sleep onset soon; having dream-like thoughts	7
Asleep	X

NASA-TLX Mental Workload Rating Scale

Please place an “X” along each scale at the point that best indicates your experience with the display configuration.

Mental Demand: How much mental and perceptual activity was required (e.g., thinking, deciding, calculating, remembering, looking, searching, etc)? Was the mission easy or demanding, simple or complex, exacting or forgiving?

Low High

Physical Demand: How much physical activity was required (e.g., pushing, pulling, turning, controlling, activating, etc.)? Was the mission easy or demanding, slow or brisk, slack or strenuous, restful or laborious?

Low High

Temporal Demand: How much time pressure did you feel due to the rate or pace at which the mission occurred? Was the pace slow and leisurely or rapid and frantic?

Low High

Performance: How successful do you think you were in accomplishing the goals of the mission? How satisfied were you with your performance in accomplishing these goals?

Low High

Effort: How hard did you have to work (mentally and physically) to accomplish your level of performance?

Low High

Frustration: How discouraged, stressed, irritated, and annoyed versus gratified, relaxed, content, and complacent did you feel during your mission?

Low High
